# Primary care-based interventions for post-stroke follow-up for long-term care: a systematic review

**DOI:** 10.1093/fampra/cmag058

**Published:** 2026-07-28

**Authors:** Hannah Ravenhall, Serena Sabatini, Rhiannon De Ivey, Linda Errington, Khalifa Mohammed, Rachael Raw, Emily Symington, Faye Tabone, Jacob Brain, Eugene Yee Hing Tang

**Affiliations:** Population Health Sciences Institute, Newcastle University, Newcastle NE2 4HH, United Kingdom; Department of Clinical Psychology and Psychobiology, University of Barcelona, Barcelona 08035, Spain; School of Psychology, University of Surrey, Guildford GU2 7XH, United Kingdom; Population Health Sciences Institute, Newcastle University, Newcastle NE2 4HH, United Kingdom; Faculty of Medical Sciences, Newcastle University, Newcastle upon Tyne NE2 4HH, United Kingdom; Population Health Sciences Institute, Newcastle University, Newcastle NE2 4HH, United Kingdom; Faculty of Medical Sciences, Newcastle University, Newcastle upon Tyne NE2 4HH, United Kingdom; Population Health Sciences Institute, Newcastle University, Newcastle NE2 4HH, United Kingdom; Nuffield Department of Clinical Neurosciences, University of Oxford, OX3 9DU, United Kingdom; Institute of Mental Health, School of Medicine, University of Nottingham, Nottingham NG7 2TU, United Kingdom; Freemasons Centre for Male Health and Wellbeing, Adelaide University, Adelaide, South Australia 5000, Australia; Population Health Sciences Institute, Newcastle University, Newcastle NE2 4HH, United Kingdom

**Keywords:** stroke, primary care, continuity of care, healthy aging, managed care

## Abstract

**Background:**

Life after stroke is managed by primary care to prevent recurrent stroke and ensure that stroke survivors live well within the community. Unfortunately, stroke survivors do not always receive timely follow-up from clinical services, which leads to unaddressed and hidden unmet needs. It is not clear what follow-up interventions would be helpful for stroke survivors in the community once discharged from specialist services.

**Aim:**

The aim of this systematic review was to investigate current pathways and interventions used in primary and community care to support patients in their lives after stroke.

**Methods:**

The review was registered on PROSPERO (CRD42024575490). Four databases were searched (MEDLINE, Embase, CINAHL, and The Cochrane Library) from 2008 to July 2024. All study types were eligible and included if based around a review of a stroke patient’s care and delivered within primary care and community settings. The Effective Practice and Organisation of Care (EPOC) taxonomy was used to classify the interventions, and the outcomes were synthesized.

**Results:**

The initial search identified 17 893 articles; 12 articles were included. These were categorized as follows: (i) coordination of care and management of care process (shared decision making *n* = 4, teams *n* = 3, and disease management *n* = 1); (ii) who provides care and how the healthcare workforce is managed (self-management, *n* = 2); and (iii) information and communication technology (smart home technologies, *n* = 2).

**Conclusion:**

Despite the current evidence, more focus is required particularly to target the hidden and unmet emotional and mental health needs of stroke survivors. The multidisciplinary nature of primary care can help to provide this holistic care.

Key messages

- Advances in medicine, education, and understanding of stroke enable more people to survive a stroke and consequently return to live in the community.
- Previous literature details the need for developments in primary and community care follow-ups to adequately address the needs of post-stoke patients.
- This review has collated current interventions and demonstrated that there is still a requirement to further development and ensure effective implementation of such holistic interventions. By doing so, it is hoped that post-stroke patients would be sufficiently supported once discharged from specialist services.

## Introduction

Over 1.3 million people in the UK are living in the community with deficits caused by stroke [1]. Ongoing care following the acute phase and rehabilitation is guided by the National Institute for Health and Care Excellence (NICE) guidelines [2]. Following discharge from stroke services, primary and community care colleagues are responsible for the ongoing management during this chronic post-stroke phase. Despite national guidance and annual reviews conducted in primary care (incentivized through Quality and Outcomes Framework (QoF) indicators [3]), stroke survivors consistently report unmet needs following discharge from hospital and/or specialist services [4].

Due to the chronic nature of post-stroke conditions, long-term structured follow-up is essential in the management of stroke survivors once discharged into the community [5, 6] Stroke can have a significant impact on the individual, causing difficulties with cognition [7], motor function and speech [8], alongside the increased risk of recurrent stroke [9]. Beyond its physical impact, stroke can profoundly affect mental health, with anxiety and depression widely reported among stroke survivors [8]. Up to one-third of patients are affected by depression within the first 12 months post-stroke [10, 11].

National guidance, for example in the UK, dictates that there should be 6-week, 6-month, and annual post-stroke follow-up [12]. Unfortunately, national audit data from the Sentinel Stroke National Audit Programme (SSNAP) revealed that from April 2023 to March 2024, only around one-third of eligible stroke survivors attend a 6-month follow-up [13]. Further, there is significant variation in how 6-month reviews are delivered in England [14]. The current annual primary care review for stroke survivors is often dictated by QoF, focusing on secondary prevention targets [3]. Other chronic conditions such as asthma and diabetes tend to have well-defined pathways for structured follow-up, but this is not always the case for stroke in primary care [15–17]. In general, post-stroke management tends to involve secondary prevention measures and reviews of adequate blood pressure (BP) and lipid control. However, the chronic nature of stroke does mean that there are often hidden effects post-stroke that will not be captured by these reviews. In 2011, Allison *et al.* [18] reviewed evidence on the effectiveness of primary care-based follow-ups and concluded that evidence is limited by the low quality and lack of theoretical basis of the included papers. This highlighted the need for high-quality research studies in the post-stroke phase [18]. Since this review, several national clinical guidelines have been developed worldwide, including the National Clinical Guideline for Stroke [19] and the NICE Stroke Rehabilitation in Adults [2] in the UK; the Secondary Stroke Prevention Guideline [20] and specific primary and community care-based guidance [8] in the USA; and Canadian guidelines [21]. This increase in evidence base may have led to the development of further primary care-based interventions and models of care that require additional synthesizing of the evidence following this initial review [18, 20].

This review therefore aims to investigate current interventions used in primary and community care, which support stroke survivors after discharge from specialist services. By looking specifically at the outcomes reported, we will be able to highlight any gaps in the holistic care of the patient.

## Methods

**Figure 1:**
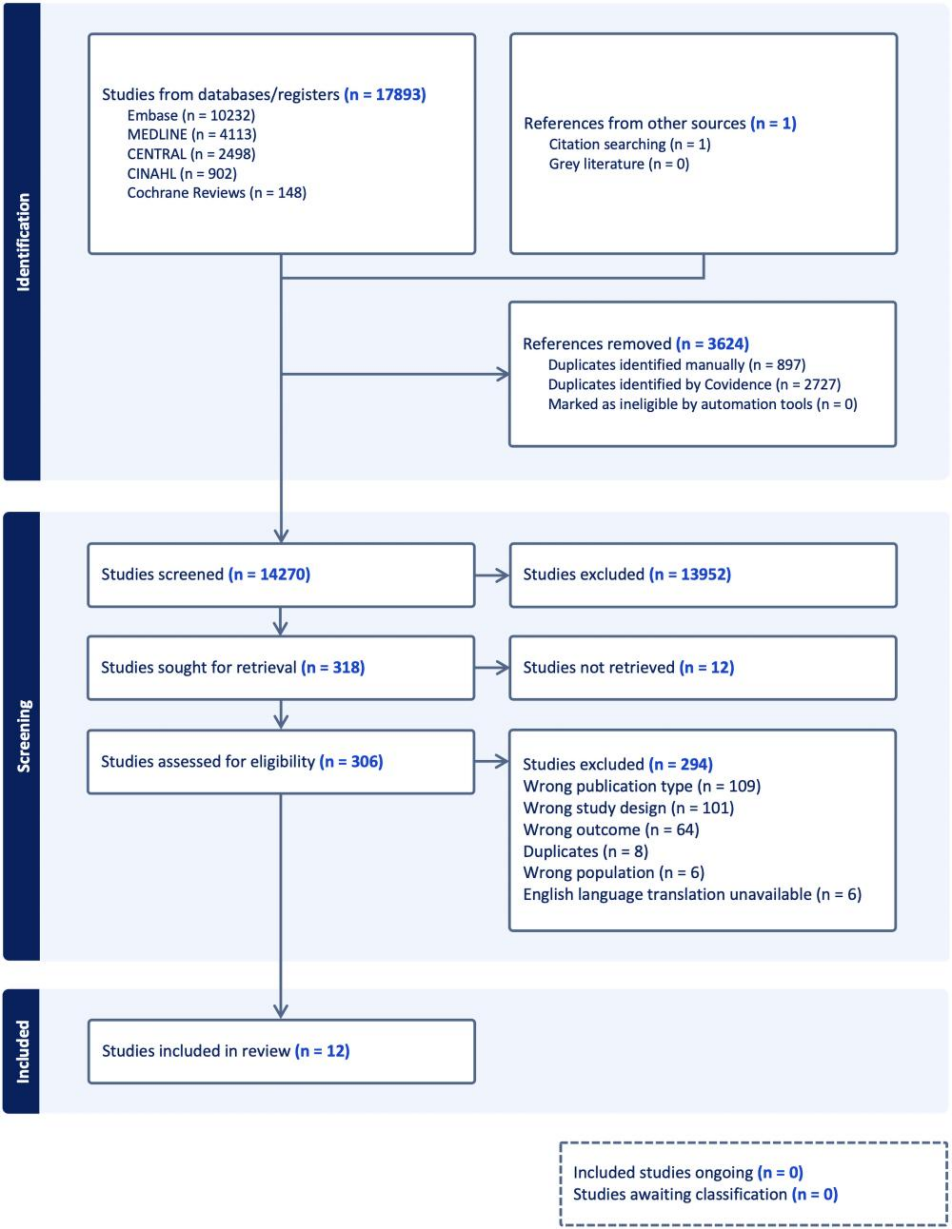
PRISMA flow diagram.

This review was registered with PROSPERO (CRD42024575490). PRISMA (Preferred Reporting Items for Systematic reviews and Meta-Analyses) guidelines were followed throughout the review (Fig. 1) [22].

### Eligibility criteria

The Population, Intervention, Comparison, and Outcome (PICO) framework was used to define the study’s eligible criteria (Box 1). With regards to study design, we were broad with our inclusion criteria to incorporate a range of study designs to ensure all relevant studies were assessed. Examples of designs included were cross-sectional, mixed methods, and non-randomized designs.

Box 1 PICO

**Table:** 

	Inclusion	Exclusion
**Population**	Individuals who have had a stroke over the age of 18 years	Individuals who have hada stroke from age 17 years and below
**Intervention**	Intervention (all study types) based in primary^a^ or community^b^ care around a review of a stroke patient’s overall health and social care which can be tied to either a post-discharge, ongoing or annual review of care.	Studies detailing interventions in secondary care or participants recruited from secondary care. Multiple population studies where specific stroke findings were not discriminated. Interventions delivered by community-based rehabilitation programmes (including community stroke services), as this would be within a specialist setting.
**Comparator**	None	N/A
**Outcome**	Measured outcomes as defined by the study	N/A

PICO, Population, Intervention, Comparison, and Outcome framework.

^a^Primary care has been defined as the first point of contact for an individual which has health concerns in the study’s healthcare setting.

^b^Community care has been defined as the care provided to enable independence for post-stroke patients, ensuring they can live at home safely, where possible. Community care services and models include, but is not limited to, the distribution of community nurses, district nurses, case managers, and social care workers.

### Search strategy

The following databases were searched: MEDLINE (OvidSP), Embase (OvidSP), CINAHL (EBSCOhost), and The Cochrane Library. We updated the search strategy from the date reported in the review by Allison *et al.* [18] (i.e. from 2008) to include studies published until July 2024. Primary research articles of any language were included. The following search terms were included: (stroke or cerebrovascular or CVA or CVE or Vascular accident) and (Long-term health or chronic disease or primary health care or family practice or community health services or follow-up).

### Selection process

An information specialist (L.E.) conducted the searches and uploaded the title/abstracts and full texts to Rayyan (systematic review management platform). After deduplication, titles and abstracts were independently screened and reviewed against PICO criteria. H.R. reviewed all titles and abstracts as a first screener, and the second screen was divided between four other authors (E.S., R.R., S.S., and F.T.). Four independent investigators (H.R., S.S., R.D.I., and K.M.) reviewed full texts to determine the final sample with all articles being double screened. A senior author (E.Y.H.T.) resolved any discrepancies where conflicts were not resolved at both stages. Reference lists of included articles were reviewed to identify any relevant studies which had been missed from the initial search.

### Data extraction

A predetermined extraction table was developed in Microsoft Excel. Data extracted included first author and year, country of origin, study design, intervention, number of participants, study setting, follow-up duration, research focus, summary of key findings, measures of impact of the intervention, and quality rating. Two investigators (H.R. and S.S.) extracted data independently before collating their findings, and any discrepancies were resolved through discussion or reviewed by a third investigator (E.Y.H.T.).

### Quality appraisal

To assess the methodological quality of the included studies, we adopted the approach outlined in Giddings *et al*.’s recent systematic review [23]. The relevant criteria were integrated from the Mixed Methods Appraisal Tool (MMAT) and the Critical Appraisal Skills Program (CASP) to accommodate the diverse range of study designs. The resulting quality appraisal framework consisted of five key criteria, each rated as green (high quality), amber (moderate quality), red (low quality), or not applicable (see Supplementary Table S1). For mixed-methods studies, an additional quality appraisal checklist was employed to ensure a comprehensive evaluation. One reviewer (J.B.) conducted the quality appraisal according to the checklist (see Supplementary Table S2). Economic evaluations were assessed exclusively using the CASP checklist for economic evaluations, as the MMAT does not provide an appraisal framework for this study type.

### Synthesis of the data

Given the heterogeneity of the included studies, a meta-analysis was not practical, therefore a data synthesis was conducted based on the Synthesis Without Meta-analysis (SWiM) in systematic reviews [24]. This approach was chosen due to the heterogeneity not only in terms of the types of interventions used but also the variation in healthcare systems and professionals used to carry out the interventions. We firstly classified the interventions based on the EPOC taxonomy: (i) delivery arrangements; (ii) financial arrangements; (iii) governance arrangements; and (iv) implementation strategies [25]. As the outcome measures varied, there was no standardized metric for each outcome reported. Instead, we summarized the direction of the effect for each intervention within their EPOC groupings. The extracted data were synthesized using descriptive and narrative techniques to assess for any emerging themes and patterns in the data linked within the individual interventions and the associated outcomes/settings.

The outcomes were then also evaluated separately and classified depending on which interventions identified unmet needs, addressed physical and/or mental health outcomes in a descriptive way (see Table 2).

## Results

Figure 1 outlines the PRISMA flow diagram. In total, 17 893 studies were identified; 318 were selected for full-text review, of which 12 met the inclusion criteria.

### Study characteristics

Table 1 details the characteristics of included studies. Most studies were conducted in the UK (*n* = 4)[5, 26–28], followed by China (*n* = 2)[29, 30], the remaining studies were from six different countries [31–36] (see Table 1). The number of participants in the studies varied from 42 to 1299. There was variation in post-stroke follow-up periods as most were tailored to the study, however a 6-month follow-up was most common [5, 27, 34, 36]. Study settings varied from telephone calls [31, 35], face-to-face appointments [26–34, 36], and home visits [29, 30, 35]. A range of study design was evaluated, including qualitative and mixed-methods designs (*n* = 6) and single-method quantitative appraisal tools (*n* = 6), which encompassed non-randomized studies (*n* = 2) and a randomized controlled trial (RCT) (*n* = 1).

**Table 1:** Summary of results.

First author, year	Country of origin	Study design	Intervention	Number of participants	Study setting	Follow-up duration	Research focus	Summary of key findings	Measures of the impact of intervention
Coordination of care and management of care process									
Shared decision making									
Iosa, 2017 [31]	Italy	Cross-sectional	PSC	64 stroke survivors	Face-to-face in GP practice or telephone interview.	N/A	PSC already utilized in the UK and Singapore, so wanted to determine the usefulness of an online PSC to assess the needs of community-dwelling Italian stroke patients	Online 11-item checklist. Identified mobility as most frequent issue, closely followed by altered mood post-stroke. Online tool increasing accessibility in the community.	PSC improved communication in 93.7% of cases. 90.6% of cases highlighted an unmet need.
Turner, 2019 [27]	UK	Mixed methods using qualitative focus groups, feasibility, and descriptive study designs	PSC	38 participants Group 1: Healthcare providers (*n* = 19) Group 2: Stroke survivors (*n* = 12) and carers (*n* = 7)	GP practice Practice nurse trained to use checklist.	The feasibility study lasted 6 months. Reviews conducted at 6–12 months post-stroke. Feedback questionnaire completion at 0 and 3 months by patient.	Feasibility of a PSC for primary care use. Qualitative analyses of focus groups to draw common themes.	At baseline, 10/12 stroke survivors felt that the PSC would be helpful in identifying needs. One participant did not complete the 3-month follow-up review. No participants correctly understood how to rank needs. Adapted 11-item checklist into a 15-item checklist. Feasibility and aspects of the intervention were amended including removal of ranking of checklist items, paperwork completion by staff, and the requirement to complete the checklist during the review if not pre-completed. Additions made to the intervention included training nurses on time management, provision of clear instructions on use of the checklist, and addressing patients about research.	Most (10/12) stroke survivors felt the checklist was easy and useful to complete. Seven stroke survivors were quite/very satisfied with the review at the 3-month follow-up. At 3-month follow-up, five stroke survivors felt that the overall care had somewhat improved. Six felt that the care had stayed the same. Eight stroke survivors would like the review to become part of routine care.
Ward, 2014 [28]	UK	Mixed methods using qualitative and descriptive cross-sectional study designs	PSC	42 participants	Face-to-face in UK general practice or hospital stroke unit.	N/A	Feasibility of the PSC was assessed by examining time taken to complete and frequency of responses. Patient understanding of the PSC was assessed qualitatively, measuring consistency of each item between clinicians and patients. Issues with the PSC were typically identified through observer notes and the cognitive debriefing interviews. Satisfaction with the PSC was assessed by clinician and patient-completed satisfaction surveys.	An 11-item checklist with yes/no answer. The most frequently identified issues in the UK cohort were cognition (75.6%), mood (73.2%), and life after stroke (70.7%). An average of five problems per patient were identified. The identified needs were consistent across UK and Singapore. Items of the checklist were well understood by patients.	High satisfaction ratings for both patients and clinicians reviewing the PSC in identifying long term stroke care needs.
Kjork, 2022 [33]	Sweden	Cross-sectional	PSC	49 participants Recruited from 20 nursing homes	Face-to-face interviews in the nursing home. Conducted with staff members or researchers.	N/A	Primary outcome: Identify health problems using a PSC. Barthel Index tested independence in personal self-care. mRS used to measure degree of disability.	Checklist items included ADLs, mobility, spasticity, pain, incontinence, communication, mood, cognition, life after stroke, and relationships with family members. Issues around ADLs were most reported, closely followed by mobility and cognition. Reduced follow-up in nursing homes where at least one in five residents had suffered a stroke; 87% of staff recommended the checklist.	PSC can support care staff in identifying the needs of residents. The results of the checklist showed that half the residents had more than six health problems each. 86% of residents with an issue in ADLs or mobility identified through the PSC were later assessed by rehabilitation professionals to target their needs. Follow-up routines in nursing home settings could be improved quite readily.
Teams									
Aziz, 2013 [32]	Malaysia	Prospective observational study	Primary care-based follow-up	91 patients	Prospective observational study of stroke patients treated at a primary care teaching facility.	1 year of follow-up with patients reviewed every 3–4 months minimum. Overall patients monitored for 2 years at the clinic.	Clinical outcomes measured included BP, lipid profile, depression (PHQ9), and Barthel Index (functional status).	Improvement in systolic BP at 1-year follow-up (55.9% vs 82.3% had a BP ≤140/90 mm Hg). Significant improvement in functional ability (using the Barthel Index). Identified a 4-month delay between acute stroke episode and contact with primary care provider. No significant improvements in lipid readings or depression.	Post-stroke management at community level yields favourable long-term outcomes for those living at home. Identified a need for secondary prevention due to high prevalence of hypertension and diabetes.
Verberne, 2020 [34]	The Netherlands	Quantitative non-randomized study	Nurse-led aftercare in supporting long-term psychosocial outcome of stroke patients	444 patients: 87 in intervention arm and 363 in the control arm (gold-standard care)	Primary Care Plus centre—45-min consultation with nurse.	6 and 12 months	Primary outcome: Analyse whether nurse-led stroke aftercare improves long-term psychosocial outcomes post-stroke. Tools used included: HADS FSS CLCE-24 USER-P SA-SIP30 EuroQoL-5D-3L	Nurse-led questionnaire around mental health, cognition, fatigue, socialization, and ADLs. Emotional problems and severity of anxiety decreased significantly after intervention. No significant difference in depressive symptoms, fatigue, and cognitive problems in daily life. Control group had a significant decrease in restrictions with socialization compared to the intervention group.	Nurse-led post-stroke care was beneficial in addition to routine follow-up stroke care. Did not show benefit for psychosocial outcome or depressive symptoms.
Egan, 2015 [35]	Canada	Mixed methods using qualitative and pre- and post-test evaluation	Community stroke navigation service	61 participants: 35 stroke survivors and 26 care partners	Intervention delivered by an OT in the community. Phone calls between visits.	One to eight visits during a 4-month period. Participants were assessed at the end of the 4 months.	Describe and evaluate a community stroke navigation programme using the following tools: HADS General Well-Being Schedule RNLI	OT-led services offered included care coordination, client and family support, just-in-time education, coaching, accompaniment, and advocacy. Time pressures on primary care physicians can impact their ability to provide navigation of services. This study supports the integration of another professional to assist with navigation, to improve accessibility.	Significant impact and improvement in community reintegration of stroke survivors but not of their care partner. No alteration in physical and emotional health among stroke survivor or care partners.
Mullis, 2024 [5]	UK	Two-arm cluster randomized control trial	IPCAS trial—GP-based service model to support stroke survivors in the long term	1040 participants: 46 GP practices (23 GP practices per arm): 522 in intervention arm and 518 in control arm	Intervention delivered at GP level.	Baseline, and 6 and 12 months	Primary outcome: development of a primary care model. Measure co-primary end points on two subscales—emotion and participation of the Stroke Impact Scale (v3.0) at 12 months after randomization. Secondary outcomes: Stroke Impact Scale–Short Form EuroQol-5-Dimension, five-level questionnaire ICEpop CAPability measure for Adults Southampton Stroke Self-Management Questionnaire Health Literacy Questionnaire	At 12 months, the intervention was not associated with any significant change in either of the coprimary outcomes: 0.08 (97.5% CI: −2.3 to 2.5) in the emotion outcome and 1.3 (97.5% CI: −2.3 to 4.9) in the participation. The study population was split into quintiles by baseline score with a non-significant increase in either score for those in the lowest quintile (compared with control arm). No evidence of effect on any secondary outcome. No evidence attending a review or having an action plan had any impact on emotional health or participation. No evidence of effect of attending the MLAS course on either outcome.	No evidence to support that this primary care model was effective in addressing the long-term needs patients. More intensive interventions focusing on patients earlier in their stroke journey may be more effective. Mismatch between needs reported by stroke survivors and evidence available on addressing these. 15-item checklist is practical for primary care use.
**Who provides care and how the healthcare workforce is managed**									
Self-management									
Blatchford, 2022 [26]	UK	Mixed methods including qualitative interviews and cross-sectional survey	Patient experience and participation in stroke self-management programme (MLAS)	420 invited from the intervention arm of the IPCAS trail 141 attended 103 completed the course	23 GP surgeries One-to-one or group-based sessions.	N/A	Primary outcome: Uptake of MLAS. Those declining were sent a questionnaire to ascertain why. Evaluation of self-management programmes through qualitative interviews and forms.	Those who did not attend the programme did so because they were: already recovering well, had ongoing health issues, faced logistical issues, or felt it was not appropriate for them. Positive experience for those who attended the intervention. It succeeded in addressing self-reported needs. Four key themes identified: Programme characteristics influence programme participation Programme content is relevant Perceived benefits of attendance Personal circumstance affects programme participation	Not all stroke survivors want to be involved in a self-management programme. Many people declined (228). For those which participated, they found it a beneficial tool.
Wang, 2013 [36]	Taiwan	Non-randomized quantitative study design	Community-based nursing education and rehabilitation programme for patients with mild stroke	127 stroke patients: 65 stroke patients allocated to the intervention arm and 62 stroke patients allocated to the control arm (gold-standard care)	Lectures, seminars, and community group sessions.	3 × 2-hour stroke interventions for 8 weeks. Assessed at baseline, 3 months, and 6 months.	Measure of knowledge, behaviour, and self-efficacy. Knowledge tested through 16 true/false/unsure questions. Twelve questions were used to assess behaviour through a 5-point strongly agree/disagree scale. The same scale was used to test self-efficacy through six items.	Stroke education sessions consisting of lectures on warning signs, clinical manifestations, risk factors, diet, social activities, and rehabilitation. Communication seminars allowed peer-teaching. Group sessions had professionals present to support survivors. Control group received hospital-based post-stroke education and rehabilitation programme.	Intervention group had significantly higher knowledge score in warning signs and medical treatment, risk factors, and dietary factors. Only social participation showed a significant change amongst behaviour scores in the intervention group.
**Information and communication technology**									
Smart home technologies									
Gong 2021 [29]	China	Quantitative non-randomized study design	Mobile App—SINEMA programme	637 patients from 25 rural villages of northern China	Face-to-face monthly visits to participants with support from an android-based mobile app. Daily voice messages (primary care based mobile intervention).	Monthly follow-up for 12 months	Primary outcome: Evaluate the implementation of a mobile app to rural Chinese villages. Use of the evaluation framework RE-AIM. Facilitators and barriers that may influence the RE-AIM dimensions were identified. A self-administered survey among village doctors was used. Survey provided to recruited participants. Monitoring data from a digital health system. Semi-structured interviews among stakeholders.	Reached a cohort which previously may have been unable to access health care. This brought significant benefits to health and well-being of participants. Components of the SINEMA app include capacity building, task shifting, home-based follow-up visits, and technology-enabled tools. Systolic BP significantly decreased. Improved medication adherence, physical activity, and quality of life. Reduced stroke recurrence, hospitalization, and death. The reach was 70% (median) of stroke survivors identified in villages. Most village doctors and participants expressed their willingness to continue the programme during interview, reporting increased confidence in prescribing evidence-based medicines and supporting patients.	Family caregivers played the most critical role in day-to-day life and treatment adherence of participants. Implementation of the SINEMA programme showed improvement, particularly relating to medication adherence and quality of life.
Yan, 2021 [30]	China	Randomized controlled trial	Mobile App—SINEMA programme	1299 participants: 611 in intervention arm; 615 in control arm; 43 lost to follow up; and 30 deaths	Face-to-face monthly visits to participants with support from an android-based mobile app. Daily voice message (primary care setting)	Monthly follow-up for 12 months	Primary outcome: 12-month change in systolic BP. Secondary outcomes: Diastolic BP HRQoL assessed by EuroQoL-5D-5L Physical activity level Self-reported medication adherence Performance in the Timed Up and Go test.	Village doctors received training, conducting monthly follow-up visits supported by android-based mobile app. They received performance-based payments. Participants received one daily voice message.	The intervention group saw a significant reduction in systolic BP with a significant improvement all secondary outcomes excluding medication adherence. This was found to be a low-cost intervention.

ADLs, activities of daily living; BP, blood pressure; CI, confidence interval; CLCE-24, Checklist for Cognitive and Emotional Consequences following Stroke; EuroQoL-5D-5L, EuroQol 5-Dimension, 5-Level Questionnaire; FSS, Fatigue Severity Scale; GPs, general practitioners; HADS, Hospital Anxiety and Depression Scale; HRQoL, Health-Related Quality of Life; IPCAS, Improving Primary Care After Stroke; MLAS, My Life After Stroke; mRS, Modified Rankin Scale; OT, occupational therapist; PHQ9, Patient Health Questionnaire-9; PSC, Post-Stroke Checklist; RE-AIM, Reach, Effectiveness, Adoption, Implementation, and Maintenance; RNLI, Reintegration to Normal Living Index; SA-SIP30, Stroke-Adapted Sickness Impact Profile; SINEMA, System-Integrated and Technology-Enabled Model of Care; USER-P, Utrecht Scale for Evaluation of Rehabilitation-Participation.

### Interventions

The 12 studies were assessed and broadly classified according to the EPOC taxonomy:

1. Coordination of care and management of care process [shared decision making (*n* = 4), teams (*n* = 3), and disease management (*n* = 1)]
2. Who provides care and how the healthcare workforce is managed [self-management (*n* = 2)]
3. Information and communication technology [smart home technologies (*n* = 2)]

### Coordination of care and management of care process

#### Shared decision making

The majority of included studies utilized Post-Stroke Checklist (PSC) as their only intervention (*n* = 4)[27, 28, 31, 33]. The checklists contained 11–15 items and sought to analyse health and well-being in broad areas such as relationships and daily functioning. Two studies used the same 11-item checklist which measured: secondary prevention, activities of daily living (ADLs), mobility, spasticity, pain, incontinence, communication, mood, cognition, life after stroke, and family relationships [28, 33]. Iosa *et al.* [31] measured intimate relations instead of ‘life after stroke’. The IPCAS (Improving Primary Care After Stroke) trial [5] was the only study to use the 15-item checklist, refined and adapted by Turner *et al*. [27]. Nine similar features included secondary prevention, ADLs, mobility, pain, incontinence, mood, communication, cognition, and family relationships. New items included stiffness, work, fatigue, social activities, intimate relations, and a final question regarding any patient concerns [5, 27]. Overall, the PSCs were found to be a useful tool in identifying the unmet needs of stroke patients [28, 31, 33]. Turner *et al.* reported that 73% of stroke patients would like a PSC component to their routine post-stroke care [27]. Based upon identification of such unmet needs, it allowed clinicians to adopt a patient-centred approach to their subsequent care. For example, Kjork *et al.* found that 85% of patients reporting an issue with ADLs through the PSC, later had a rehabilitation assessment to address such needs [33].

#### Teams

Three studies detailed interventions consisting of follow-up with healthcare professionals [32, 34, 35]. A multidisciplinary team approach within a primary care setting was used by Aziz *et al.*, comprising consultants, nurses, and a clinic aide [32]. Expert follow-up allowed secondary prevention measures to be implemented and monitored, and alongside community rehabilitation, improved the long-term care of stroke survivors [32]. Implementation of a structured care plan resulted in a significant drop in BP and improvement in functional ability (measured by Barthel Index) at 1-year follow-up [32]. A reduction in the Patient Health Questionnaire-9 (PHQ-9) scores at 1-year follow-up suggests a decrease in depressive symptoms post-intervention [32]. Verberne *et al.* described a nurse-delivered intervention which aimed to improve psychosocial well-being of stroke survivors [34]. Patients received questionnaires assessing emotional and psychosocial outcomes [34]. These were used to inform the nurse of any patient concerns prior to consultation, allowing psychoeducation, support, and/or referrals to be implemented [34]. The nurse-led intervention resulted in a significant decrease in anxiety and emotional problems in the intervention group [34]. Egan *et al.* [35] delivered their tool through an occupational therapist (OT), which supported stroke survivors to access different community-based health care services [35]. The OT navigated patients and their caregivers towards services tailored to their needs (e.g. case coordinators, coaching, and education) [35]. There was improvement in walking ability and mood among stroke survivors and their caregivers following the intervention [35]. It was evidenced that the tool significantly improved community reintegration as supported by a significant Reintegration to Normal Living Index [35].

#### Disease management

The IPCAS trial consisted of multicomponent intervention, which included: (i) a review of stroke-related needs; (ii) a self-management programme; (iii) a direct point of contact in general practice; (iv) enhanced communication between care services; and (v) a directory of national and local community services [5]. Although this programme reaffirmed the need for long term interventions to support stroke survivors, the intervention itself was not effective in addressing these needs. Earlier or more intensive interventions may be more effective, and there was a mismatch between the reported needs and the available evidence to address them [5].

### Who provides care and how the healthcare workforce is managed

#### Self-management

The My Life After Stroke (MLAS) programme (an arm of IPCAS trial [5]) provided evidence to support the use of a self-management programme in the aftercare of stroke survivors [26]. The programme consisted of individual appointments and group sessions with different focusses, managing health and well-being, ‘roadblocks’, and moving forward with their journey [26]. The sessions supported the understanding of an individual’s stroke journey and looked at ways to prevent future strokes in the individual. The MLAS programme helped survivors to identify risk factors, whilst recognizing the importance of physical activity and an awareness around their own post-stroke barriers [26]. The evaluation showed that participants found the programme useful and that it addressed intended principles [26].

Wang *et al.* described a community-based education programme for survivors, which delivered stroke education sessions and patient support groups [36]. Healthcare professionals (e.g. therapists and nurses) were also present to enhance the support provided to stroke survivors [36]. The educational sessions were delivered by lectures and demonstrations to discuss risk factors, warning signs, clinical manifestations, and social activities [36]. At 6-month follow-up, the intervention group was found to have significant improvements around the understanding of warning signs, risk factors, and dietary factors contributing to stroke recurrence [36]. A significant improvement in social participation post-stroke was found following educational input [36].

### Information and communication technology

#### Smart home technologies

Gong *et al.* [29] and Yan *et al.* [30] both detailed the use of a mobile-health intervention to improve post-stroke care for residents in Chinese villages. Yan *et al.* [30] discussed the implementation of the intervention, with Gong *et al.* [29] evaluating the effectiveness of implementation of the intervention. A mobile application was used to send daily voice messages to patients, which were supported by monthly follow-up visits from village doctors [29, 30]. Daily voice messages consisted of reminders to take their daily medications and do some physical activity, with over 180 co-designed message options [29, 30]. The intervention saw a significant improvement in BP, health-related QoL, physical activity, mobility functioning, and adherence to statins and antihypertensives for the stroke survivors.

### Intervention outcomes reported

Due to the heterogeneous nature of both the interventions and the outcomes measured, it was not possible to perform a meta-analysis. We have tabulated and summarized the intervention outcomes and classified them according to three separate categories namely interventions, which sought to address (i) an unmet need identified by the stroke survivor, (ii) a physical health outcome, and (iii) a mental health outcome including cognition (see Table 2). There are generally high satisfaction levels from participants when a person-orientated intervention such as a PSC is used to identify an unmet need. Generally, when physical health measures such as mobility, function, and BPs were measured, these were found to have improved. The majority of studies reporting on mental health outcomes did not demonstrate an improvement.

**Table 2:** Reported outcomes by EPOC taxonomy.

Intervention focus	Identifying unmet need	Physical health outcome	Mental health outcome
Coordination of care and management of care processes	Five studies [5, 27, 28, 31, 33] had the aim in identifying the unmet needs of stroke patients through the use of a PSC. High satisfaction rates in identifying unmet needs [28, 31, 33] [27], with one study [5] unable to evidence the benefit of a primary care-based model in effectively addressing the long-term needs of stroke patients.	Four studies [27, 31–33] found that physical health had improved due to the intervention with mobility one of the most frequent issues identified [27, 31, 33]. One study [32] found an improvement in blood pressure control and functional ability at 1-year follow-up through regular (every 3–4 months) primary care reviews. One study found that an occupational therapist-led community stroke navigation programme found some improvement in walking ability (yet not significantly) [35].	A nurse-led model of post-stroke care [34], found a significant reduction in emotional problems and severity of anxiety. Three studies found no significant improvement in mental health through their intervention [5, 32, 35]
Who provides care and how the healthcare workforce is managed	One study found that a self-management tool was beneficial in identifying the needs of stroke survivors who participated in the programme [26].	A nursing education and rehabilitation programme found a significant improvement in stroke knowledge, for example stroke-related risk factors, dietary factors, and warning signs to prevent recurrent stroke and therefore enabling optimal physical health management post-stroke [36].	N/A
Information and communication technology	N/A	A mobile app-based programme found a significant improvement in blood pressure, medication adherence, physical activity, and health-related quality of life [29, 30].	N/A

EPOC, Effective Practice and Organisation of Care; N/A, not applicable; PSC, Post-Stroke Checklist.

### Quality appraisal

Of the 12 included studies, 6 employed mixed-methods designs and were assessed using both qualitative and mixed-methods appraisal checklists. These studies incorporated a range of quantitative methodologies, including one RCT (*n* = 1) [5], two non-randomized studies (*n* = 2) [29, 35], and three descriptive studies (*n* = 3) [26–28]. The remaining six studies were evaluated using single-method quantitative appraisal tools, including the RCT checklist (*n* = 1) [30], non-randomized study checklist (*n* = 3) [32, 34, 36], and quantitative descriptive checklist (*n* = 2) [31, 33] (see Supplementary Table S1).

The mixed-methods studies demonstrated strong methodological quality overall, with RCT-based designs scoring highly in randomization and statistical analysis, although allocation concealment remained a moderate concern [5]. Among non-randomized mixed-methods studies, confounder control was a key limitation, potentially introducing selection and measurement biases. Descriptive mixed-methods studies exhibited moderate concerns related to recruitment and nonresponse bias, although they demonstrated strong methodological integration. Across all mixed-methods studies, better alignment between quantitative and qualitative findings, alongside stricter adherence to methodological rigour for each study component, would enhance their validity. One study [5] performed an economical evaluation, scoring ‘Yes’ across questions 1–11; on question 12, it could not be determined whether the findings could be implemented in a different setting. For full details, see Supplementary Tables S1 and S2.

Among the six single-method quantitative studies, the only study, which was the RCT, scored high across all domains except allocation concealment [30]. The non-randomized studies [32, 34, 36] were of moderate quality, with strong research design and outcome reporting, but limitations in recruitment representativeness and confounder adjustment, potentially introducing bias. The quantitative descriptive studies [31, 33] demonstrated gaps in recruitment strategy and interpretation of findings, with neither study reporting nonresponse rates.

## Discussion

Overall, this review demonstrates that there has been an increase in the innovative ways to assist primary care clinicians in managing patients holistically in the post-stroke phase. However, it is clear that whilst interventions to improve physical health indices and outcomes has had positive outcomes, the interventions are not as effective for the hidden effects post-stroke, namely mental health and emotional difficulties.

This review serves as a more inclusive review of the available evidence compared to the 2011 review [18], which detailed nine RCTs based upon primary care interventions for stroke survivors. It was found that no sound theoretical basis was detailed for the interventions, which could have contributed to the inadequate implementation and poor communications between clinicians and survivors mentioned [18]. Since then, the IPCAS trial is the most complete programme aimed at improving primary care after stroke [5]. Aspects of this trial have already been discussed [26, 27] and demonstrate the need to support stroke survivors once discharged from specialist care. Unfortunately, this multicomponent approach did not adequately address the needs of stroke survivors and, similar to other studies, had no significant benefit on mental health outcomes. Mental health and emotional and cognitive problems are the top two research priorities identified by key stakeholders [37]. Despite this, we are currently lacking an evidence base within primary care to address this.

One challenge in addressing post-stroke needs is the heterogeneity across individuals and the implementation of a cohesive and comprehensive PSC to help clinicians identify specific needs. In general, PSCs seem to be well received in the included studies for this review. This has been highlighted for example as part of routine specialist care, where an intervention used at 6-month reviews is the Greater Manchester Stroke Assessment Tool (GM-SAT). When used in the community, the GM-SAT demonstrated feasibility and acceptability with both patients and carers [38]. This feasibility extends to individuals residing in care homes [39]; whereby there may be additional challenges in attending a 6-month review at a specialist clinic. However, simple identification is insufficient; it must be coupled with clear patient benefits by either reducing the impact of post-stroke difficulties or the risk of recurrent stroke. This could be achieved through improved integration of specialist, primary and community care services as seen in other chronic conditions [40]. This was demonstrated by studies that incorporated members of the wider clinical team, either as a multidisciplinary team [32] or as individuals (nursing [34] and OT [35]) to address emotional difficulties and social reintegration. In particular, what is offered by the OT-based intervention in Egan *et al.*’*s* study [35] is much more readily available through social prescriber link workers in the community. Although further evidence is needed [41], particularly in the context of multimorbidity and social deprivation [42], there may be benefits in specific diseases where primary care follow-up is limited.

Dementia is another example where there are similar challenges such as social isolation, lack of tailored primary care support, and difficulties in accessing support when needed. The Social Prescribing for people to Live ENjoyably with Dementia/memory problems In Daily life (SPLENDID) programme is currently developing a novel blended approach to social prescribing in the context of dementia [43]. The holistic and personalized nature of this social prescribing approach could be complimentary to the ongoing second prevention measures used by the clinical team and could be developed with support from stroke survivors. It is also likely that some of the other hidden measures such as cognition and emotional difficulties could be picked up and addressed through this holistic approach.

Other healthcare systems are beginning to recognize the importance of holistic primary care-led chronic stroke management. One example can be found in the USA, where a patient-centred approach is followed, and patients can detail their most valued health outcomes, enabling clinicians to tailor their care [8]. The development of screening tools and goal-directed pathways can provide a consistent framework for primary care physicians to support post-stroke patients [8]. This could work in conjunction with effective self-help measures supported by assistive technologies. Given the complexity of life after stroke, interventions need to be tailored to the individual rather than a one size fits all approach.

This systematic review highlights the categories of interventions that aim to improve the long-term care of stroke survivors. Adopting a patient-centred approach, orientated around identifying patient needs whilst providing individualized education and support, is likely to be effective and beneficial in the long-term care of post-stroke patients. It is important that interventions are readily accessible to patients immediately after discharge from secondary care, rather than at planned 6-month or annual reviews. These interventions could form the components of a care offer to stroke patients at the point of transfer back into primary care following their acute event. Future research should look at ways to fully utilize the entirety of the wider primary and community care teams in delivering effective interventions whilst co-producing an effective pathway to support the stroke survivor and their families.

## Limitations

This review was inclusive and did not restrict the inclusion criteria on study design or language, which meant that we could capture innovative models of care in other settings including in low- and middle-income countries. We focused solely on primary care-based interventions and were more inclusive than a previous review in a similar area, which had also included articles that had identified their patients before discharge from hospital in RCTs [18]. We do however recognize several limitations. The heterogeneity of the included studies in this review meant that a coherent narrative was challenging. There are also recognized limitations in categorizing the interventions according to the EPOC taxonomy, including the potential for overlap across several categories. The subsequent synthesis of the outcomes were also broad due to the heterogeneity of outcome measures described. Finally, although we were clear in our definition of what constitutes primary and community care, this would depend on the healthcare setting it was based upon, making comparisons more difficult.

## Conclusion

Through different initiatives, there is potential to improve the post-stroke care of individuals once they are in the community. This review has synchronized various worldwide intervention categories which all have the potential to meet the needs of stroke survivors. One aspect that needs additional focus are interventions that address the hidden impact on emotional and mental well-being of the stroke survivor. Although much focus continues to be on physical rehabilitation and secondary prevention, all wellbeing aspects of the stroke survivor needs to be equally supported so that they can continue to live well in the lives after stroke. The next steps are to identify professionals within the multidisciplinary primary care team who can help support stroke survivors, and which interventions could be delivered in primary care to support their unmet needs.

## Supplementary Material

cmag058_Supplementary_Data

## Data Availability

The data underlying this article are available in the article and in its online Supplementary material.
