# Supplementary material for: Primary care-based interventions for post-stroke follow-up for long-term care: a systematic review
Source: Fam Pract. 2026 Jul 28;43(4):cmag058. doi: 10.1093/fampra/cmag058 (PMC13409119; doi:10.1093/fampra/cmag058)
Supplement: cmag058_Supplementary_Data [file cmag058_supplementary_data.pdf]

Supplementary Table 1: Quality Appraisal

		Question 1	Question 2	Question 3	Question 4	Question 5
	Qualitative checklist					
	Blatchford*	1	2	1	1	2
	Egan*	1	2	2	2	3
	Gong*	1	1	1	1	2
	IPCAS*	1	1	1	1	1
	Turner*	1	2	1	1	2
	Ward*	1	2	1	1	2
	Quantitative randomised					
	IPCAS*	1	4	1	2	1
Mixed-Method Studies	Quantitative non-randomised					

Egan*	2	1	2	4	1
Gong*	1	1	1	2	1
Quantitative descriptive (e.g. cross-sectional)					
Blatchford*	2	1	4	1	1
Turner*	2	1	4	1	1
Ward*	2	1	4	1	2
Mixed Methods*					
Blatchford*	1	1	1	2	2
Egan*	2	2	2	3	2
Gong*	1	1	1	1	1
IPCAS*	1	1	2	2	1
Turner*	1	1	1	2	2
Ward*	1	1	1	2	1

Non Mixed-Method Studies

Quantitative randomised					
Yan	1	2	1	1	1
Quantitative non-randomised					
Aziz	2	1	2	2	1
Verbene	2	1	2	1	1
Wang	2	1	2	2	1
Quantitative descriptive (e.g. cross-sectional)					
Iosa	2	1	4	1	2
Kjork	2	1	4	1	2

Key: High quality = Green; Moderate quality = Amber; Low quality = Red; Not Applicable = Grey

Supplementary Table 2: Quality Appraisal Checklist

Checklist	Question	Points to consider
Qualitative checklist	1. Is the qualitative approach appropriate to answer the research question?	• If the research seeks to interpret or illuminate the actions and/or subjective experiences of research participants<br>• Is qualitative research the right methodology for addressing the research goal?
	2. Was the recruitment strategy appropriate to the aims of the research?	• Explanation of how participants were selected - was random/non-random sampling employed and was this appropriate?<br>• Clear rationale as to why the

		participants they selected were the most appropriate to provide access to the type of knowledge sought. Was anyone missing?<br>• If there are any discussions around recruitment (e.g., why some people chose not to take part, inclusion/exclusion criteria)<br>• Discussion of sample size target?<br>• Do participants reflect the applicable population?<br>• Is there justification around under/over representation of groups, and is additional recruitment attempted to resolve

		this?
	3. Was the research design appropriate to address the aims of the research?	• Justification for the research design (e.g., have they discussed how they decided which method to use). Does it seem appropriate to answer the question?<br>• Explanation of how data collection was carried out (Do authors provide the questions asked - topic guide/template available?)<br>• Discussion of saturation of data<br>• Form of data clear (e.g., tape recordings, video materials notes, etc.)<br>• If methods were modified during

		the study. If so, has the researcher explained how and why?<br>• Did the researcher critically examine their own role, potential bias, and influence during (a) formulation of the research questions (b) data collection, including sample recruitment and choice of location?
	4. Are the findings adequately derived from the data?	• If there is an in-depth description of the analysis process (e.g., how did they code, who did the coding?)<br>• Did triangulation occur – e.g., more than one researcher

		performing coding?<br>• If sufficient data (quotes) is presented to support the findings<br>• To what extent is contradictory data taken into account?<br>• Whether the researcher critically examined their own role, potential bias and influence during analysis and selection of data for presentation
	5. Is there an appropriate level of interpretive rigour?	• If there is adequate discussion of the evidence both for and against the researcher's arguments

		• If the researcher has discussed the credibility of their findings (e.g., triangulation, respondent validation, more than one analyst).<br>• Are inferences consistent with other theory/state of knowledge/expert opinion?<br>• If the findings are discussed in relation to the original research question.<br>• Have they adequately taken account of all the limitations in the methods?

Randomised control studies checklist	1. Is randomization appropriately performed?	• How was randomisation carried out? Was the method appropriate? N.B. A simple statement such as 'we randomly allocated' or 'using a randomized design' is insufficient to judge if randomization was appropriately performed<br>• Was randomisation sufficient to eliminate systematic bias?<br>• Was the allocation sequence concealed from investigators and participants?

	2. Is appropriate allocation concealment performed?	• Were the participants 'blind' to intervention they were given?<br>• Were the investigators 'blind' to the intervention they were giving to participants?<br>• Were the people assessing/analysing outcome/s 'blinded'?
	3. Are study groups similar at baseline and during the study?	• Were the baseline characteristics of each study group (e.g., age, sex, socio-economic group) clearly set out?<br>• Were there any differences between the study groups that could affect the outcome/s?

		• If any additional interventions were given (e.g., tests or treatments), were they similar between the study groups?<br>• Were the follow-up intervals the same for each study group?
	4. Are there complete outcome data?	• Was the level of drop out acceptable? In the literature, acceptable complete data value ranged from 80% (Thomas et al., 2004; Zaza et al., 2000) to 95% (Higgins et al., 2016)<br>• Were losses to follow-up and exclusions after randomisation accounted for?

		• Were participants analysed in the study groups to which they were randomised (intention-to-treat analysis)?<br>• Was the study stopped early? If so, what was the reason?
	5. Are the findings adequately derived from the data?	• Was a power calculation undertaken?<br>• What outcomes were measured, and were they clearly specified?<br>• How were the results expressed? For binary outcomes, were relative and absolute effects reported?<br>• Were the results reported for each outcome in each

		study group at each follow-up interval?<br>• Was there any missing or incomplete data?<br>• Was there differential drop-out between the study groups that could affect the results?<br>• Were potential sources of bias identified?<br>• Which statistical tests were used?<br>• Were p values reported?<br>• Were confidence intervals (CIs) reported?
Non-	1. Was the recruitment strategy appropriate to	• Explanation of how the participants were selected - was

	the aims of the research?	random/non-random sampling employed and was this appropriate?<br>• Clear why the participants they selected were the most appropriate to provide access to the type of knowledge sought by the study. Was anyone missing?<br>• If there are any discussions around recruitment (e.g., why some people chose not to take part, inclusion/exclusion criteria)<br>• Discussion of sample size target?<br>• Do participants reflect the

		applicable population?<br>• Is there justification around under/over representation of groups, and is additional recruitment attempted to resolve this?
	2. Was the research design appropriate to address the aims of the research?	N.B. For time series, want to measure changes in outcomes over time (so exposure is change in use of system over time)<br>• Justification for data collection method (e.g., survey/system use etc.)/does it seem appropriate to answer the question?

		• Clarity in explanation in how data collection was carried out (Do authors provide a copy of the survey questions?)<br>• If methods were modified during the study. If so, has the researcher explained how and why?<br>• Did they use subjective or objective measurements? Do the measurements truly reflect what you want them to (have they been validated or are they 'gold standard')?
	3. Are there complete outcome data?	N.B. For time series, want to measure changes in outcomes over time

		• Was the level of drop out acceptable? in the literature, acceptable complete data value ranged from 80% (Thomas et al., 2004; Zaza et al., 2000) to 95% (Higgins et al., 2016)<br>• Were there differences in outcomes (thoughts regarding tools) to those lost to follow-up?<br>• Was follow up time appropriate to see differences over time?<br>• Was the study stopped early? If so, what was the reason?<br>• Was the intervention administered as expected

		during the study period?<br>• Were there any unplanned co-interventions present that may influence their findings?
	4. Are the confounders accounted for in the design and analysis?	Confounders predict the outcome of interest and the intervention received/exposure at baseline. They can distort the interpretation of findings and need to be considered in the design and analysis of a non-randomized study. Confounding bias is low if there is no confounding expected, or appropriate methods to control for confounders are used (such as stratification, regression,

		matching, standardization, and inverse probability weighting).
	5. Are the findings adequately derived from the data?	• Have they reported changes over time? And strength of the association between outcomes and time<br>• What outcomes were measured, and were they clearly specified?<br>• How were the results expressed?<br>• Were the results reported for each outcome in each study group at each follow-up interval?<br>• Was there any missing or incomplete data?<br>• Was there differential

		drop-out between the study groups that could affect the results?<br>• Were potential sources of bias identified?<br>• Which statistical tests were used?<br>• Were p values reported?<br>• Were confidence intervals (CIs) reported?
Quantitative descriptive studies checklist	1. Was the recruitment strategy appropriate to the aims of the research?	• Explanation of how the participants were selected - was random/non-random sampling employed and was this appropriate?<br>• Clear why the participants they selected were the most

		appropriate to provide access to the type of knowledge sought by the study. Was there anyone missing?<br>• If there are any discussions around recruitment (e.g., why some people chose not to take part, inclusion/exclusion criteria)<br>• Discussion of sample size target?<br>• Do participants reflect the applicable population?<br>• Is there justification around under/over representation of groups, and is additional recruitment attempted to resolve

		this?<br>• If no sample size calculation, max amber rating
	2. Was the research design appropriate to address the aims of the research?	• Justification for data collection method (e.g., survey)/does it seem appropriate to answer the question?<br>• Clarity in explanation in how data collection was carried out (Do authors provide a copy of the survey questions?)<br>• If methods were modified during the study. If so, has the researcher explained how and why?<br>• Did they use subjective or objective measurements?

		• Do the measurements truly reflect what you want them to (have they been validated or are they 'gold standard')?
	3. Is the risk of nonresponse bias low?	• Does the paper report nonresponse rate (if yes, amber)?<br>• Reasons for non-response (e.g., non-contacts vs. refusals), and statistical compensation for nonresponse (e.g., imputation).<br>• Are responders different to non-responders?<br>• If no mention of non-responders, then mark as NA
	4. Are the findings	• Is the choice of statistical

	adequately derived from the data?	analysis justified/sensible?<br>• Do limitations in data analysis performed lead to limited interpretation of results?<br>• Does statistical analysis enable the study question to be answered?
	5. Is there an appropriate level of interpretive rigour?	• If there is adequate discussion of the evidence both for and against the researcher's arguments<br>• If the researcher has discussed the credibility of their findings (e.g., triangulation, respondent validation, more than one analyst)

		• Are inferences consistent with other theory/state of knowledge/expert opinion?<br>• If the findings are discussed in relation to the original research question<br>• Have they adequately taken account of all the limitations in the methods?
Mixed-Methods checklist	1. Was the recruitment strategy appropriate to the aims of the research?	•Explanation of how the participants were selected - was random/non-random sampling employed and was this appropriate?<br>• Clear why the participants they selected were the most appropriate to provide access to

		the type of knowledge sought by the study. Was anyone missing?<br>• If there are any discussions around recruitment (e.g., why some people chose not to take part, inclusion/exclusion criteria)<br>• Discussion of sample size target?<br>• Do participants reflect the applicable population?<br>• Is there justification around under/over representation of groups, and is additional recruitment attempted to resolve this?

		• If no sample size calculation, max amber rating
	2. Was the research design appropriate to address the aims of the research?	• Justification for data collection method (e.g., survey)/does it seem appropriate to answer the question?<br>• Clarity in explanation in how data collection was carried out (Do authors provide a copy of the survey questions?)<br>• If methods were modified during the study. If so, has the researcher explained how and why<br>• Did they use subjective or objective measurements?<br>• Do the measurements truly

		reflect what you want them to (have they been validated or are they 'gold standard')?
	3. Is the risk of nonresponse bias low?	• Does paper report nonresponse rate (if yes, amber)?<br>• Reasons for nonresponse (e.g., noncontacts vs. refusals), and statistical compensation for nonresponse (e.g., imputation).<br>• Are responders different to non-responders?<br>• If no mention of non-responders, then mark as NA
	4. Are the findings adequately derived from the data?	• Is the choice of statistical analysis justified/sensible?<br>• Do limitations in data analysis

		performed lead to limited interpretation of results?<br>• Does statistical analysis enable the study question to be answered?
	5. Is there an appropriate level of interpretive rigour?	• If there is adequate discussion of the evidence both for and against the researcher's arguments<br>• If the researcher has discussed the credibility of their findings (e.g., triangulation, respondent validation, more than one analyst)<br>• Are inferences consistent with other theory/state of

		knowledge/expert opinion?<br>• If the findings are discussed in relation to the original research question<br>• Have they adequately taken account of all the limitations in the methods?

PRISMA 2020 Checklist

Section and Topic	Item #	Checklist item	Location where item is reported
**TITLE**			
Title	1	Identify the report as a systematic review.	1
**ABSTRACT**			
Abstract	2	See the PRISMA 2020 for Abstracts checklist.	3-4
**INTRODUCTION**			
Rationale	3	Describe the rationale for the review in the context of existing knowledge.	5-6
Objectives	4	Provide an explicit statement of the objective(s) or question(s) the review addresses.	5-6
**METHODS**			
Eligibility criteria	5	Specify the inclusion and exclusion criteria for the review and how studies were grouped for the syntheses.	7 and box 1
Information sources	6	Specify all databases, registers, websites, organisations, reference lists and other sources searched or consulted to identify studies. Specify the date when each source was last searched or consulted.	7
Search strategy	7	Present the full search strategies for all databases, registers and websites, including any filters and limits used.	7
Selection process	8	Specify the methods used to decide whether a study met the inclusion criteria of the review, including how many reviewers screened each record and each report retrieved, whether they worked independently, and if applicable, details of automation tools used in the process.	7
Data collection process	9	Specify the methods used to collect data from reports, including how many reviewers collected data from each report, whether they worked independently, any processes for obtaining or confirming data from study investigators, and if applicable, details of automation tools used in the process.	7-8
4Data items	10a	List and define all outcomes for which data were sought. Specify whether all results that were compatible with each outcome domain in each study were sought (e.g. for all measures, time points, analyses), and if not, the methods used to decide which results to collect.	8
	10b	List and define all other variables for which data were sought (e.g. participant and intervention characteristics, funding sources). Describe any assumptions made about any missing or unclear information.	8
Study risk of bias assessment	11	Specify the methods used to assess risk of bias in the included studies, including details of the tool(s) used, how many reviewers assessed each study and whether they worked independently, and if applicable, details of automation tools used in the process.	8
Effect measures	12	Specify for each outcome the effect measure(s) (e.g. risk ratio, mean difference) used in the synthesis or presentation of results.	NA
Synthesis methods	13a	Describe the processes used to decide which studies were eligible for each synthesis (e.g. tabulating the study intervention characteristics and comparing against the planned groups for each synthesis (item #5)).	7-9
	13b	Describe any methods required to prepare the data for presentation or synthesis, such as handling of missing summary statistics, or data conversions.	NA
	13c	Describe any methods used to tabulate or visually display results of individual studies and syntheses.	NA

Section and Topic	Item #	Checklist item	Location where item is reported
	13d	Describe any methods used to synthesize results and provide a rationale for the choice(s). If meta-analysis was performed, describe the model(s), method(s) to identify the presence and extent of statistical heterogeneity, and software package(s) used.	NA
	13e	Describe any methods used to explore possible causes of heterogeneity among study results (e.g. subgroup analysis, meta-regression).	NA
	13f	Describe any sensitivity analyses conducted to assess robustness of the synthesized results.	NA
Reporting bias assessment	14	Describe any methods used to assess risk of bias due to missing results in a synthesis (arising from reporting biases).	8
Certainty assessment	15	Describe any methods used to assess certainty (or confidence) in the body of evidence for an outcome.	NA
**RESULTS**			
Study selection	16a	Describe the results of the search and selection process, from the number of records identified in the search to the number of studies included in the review, ideally using a flow diagram.	10 and figure 1
	16b	Cite studies that might appear to meet the inclusion criteria, but which were excluded, and explain why they were excluded.	10 and figure 1
Study characteristics	17	Cite each included study and present its characteristics.	Table 1
Risk of bias in studies	18	Present assessments of risk of bias for each included study.	6-7 and table 2
Results of individual studies	19	For all outcomes, present, for each study: (a) summary statistics for each group (where appropriate) and (b) an effect estimate and its precision (e.g. confidence/credible interval), ideally using structured tables or plots.	Table 1
Results of syntheses	20a	For each synthesis, briefly summarise the characteristics and risk of bias among contributing studies.	14-15 and table 2
	20b	Present results of all statistical syntheses conducted. If meta-analysis was done, present for each the summary estimate and its precision (e.g. confidence/credible interval) and measures of statistical heterogeneity. If comparing groups, describe the direction of the effect.	NA
	20c	Present results of all investigations of possible causes of heterogeneity among study results.	10-15 and table 2
	20d	Present results of all sensitivity analyses conducted to assess the robustness of the synthesized results.	NA
Reporting biases	21	Present assessments of risk of bias due to missing results (arising from reporting biases) for each synthesis assessed.	14-15 and table 2
Certainty of evidence	22	Present assessments of certainty (or confidence) in the body of evidence for each outcome assessed.	NA
**DISCUSSION**			
Discussion	23a	Provide a general interpretation of the results in the context of other evidence.	16

Section and Topic	Item #	Checklist item	Location where item is reported
	23b	Discuss any limitations of the evidence included in the review.	18
	23c	Discuss any limitations of the review processes used.	18
	23d	Discuss implications of the results for practice, policy, and future research.	16-19
**OTHER INFORMATION**			
Registration and protocol	24a	Provide registration information for the review, including register name and registration number, or state that the review was not registered.	7
	24b	Indicate where the review protocol can be accessed, or state that a protocol was not prepared.	PROSPERO
	24c	Describe and explain any amendments to information provided at registration or in the protocol.	PROSPERO
Support	25	Describe sources of financial or non-financial support for the review, and the role of the funders or sponsors in the review.	20
Competing interests	26	Declare any competing interests of review authors.	20
Availability of data, code and other materials	27	Report which of the following are publicly available and where they can be found: template data collection forms; data extracted from included studies; data used for all analyses; analytic code; any other materials used in the review.	NA

From: Page MJ, McKenzie JE, Bossuyt PM, Boutron I, Hoffmann TC, Mulrow CD, et al. The PRISMA 2020 statement: an updated guideline for reporting systematic reviews. BMJ 2021;372:n71. doi: 10.1136/bmj.n71. This work is licensed under CC BY 4.0. To view a copy of this license, visit https://creativecommons.org/licenses/by/4.0/
